# New Platform Technology for Comprehensive Serological Diagnostics of Autoimmune Diseases

**DOI:** 10.1155/2012/284740

**Published:** 2012-12-19

**Authors:** Annika Willitzki, Rico Hiemann, Vanessa Peters, Ulrich Sack, Peter Schierack, Stefan Rödiger, Ursula Anderer, Karsten Conrad, Dimitrios P. Bogdanos, Dirk Reinhold, Dirk Roggenbuck

**Affiliations:** ^1^Institute of Molecular and Clinical Immunology, Otto-von-Guericke University, 39120 Magdeburg, Germany; ^2^Faculty of Natural Sciences, Lausitz University of Applied Sciences, 01968 Senftenberg, Germany; ^3^R/D, Medipan GmbH, 15827 Dahlewitz/Berlin, Germany; ^4^Institute of Clinical Immunology, Medical Faculty, University of Leipzig, 04103 Leipzig, Germany; ^5^Institute of Immunology, Technical University, 01307 Dresden, Germany; ^6^Division of Transplantation Immunology and Mucosal Biology, King's College London School of Medicine, King's College Hospital, Denmark Hill Campus, Bessemer Road, London SE5 9RJ, UK

## Abstract

Antibody assessment is an essential part in the serological diagnosis of autoimmune diseases. However, different diagnostic strategies have been proposed for the work up of sera in particular from patients with systemic autoimmune rheumatic disease (SARD). In general, screening for SARD-associated antibodies by indirect immunofluorescence (IIF) is followed by confirmatory testing covering different assay techniques. Due to lacking automation, standardization, modern data management, and human bias in IIF screening, this two-stage approach has recently been challenged by multiplex techniques particularly in laboratories with high workload. However, detection of antinuclear antibodies by IIF is still recommended to be the gold standard method for antibody screening in sera from patients with suspected SARD. To address the limitations of IIF and to meet the demand for cost-efficient autoantibody screening, automated IIF methods employing novel pattern recognition algorithms for image analysis have been introduced recently. In this respect, the AKLIDES technology has been the first commercially available platform for automated interpretation of cell-based IIF testing and provides multiplexing by addressable microbead immunoassays for confirmatory testing. This paper gives an overview of recently published studies demonstrating the advantages of this new technology for SARD serology.

## 1. Introduction

Systemic autoimmune rheumatic diseases (SARDs), such as systemic lupus erythematosus (SLE), rheumatoid arthritis (RA), systemic sclerosis (SSc), idiopathic inflammatory myopathies (IIM), Sjögren's syndrome (SjS), and antineutrophil cytoplasmic antibody (ANCA) associated systemic vasculitis (AASV), are often accompanied by the occurrence of nonorgan-specific autoantibodies (AAb) [1–4]. Especially, antinuclear antibodies (ANA) and anticytoplasmatic autoantibodies (ACyA) have been proven to be useful markers in the serological diagnosis of SARD and may also assist in the prognosis, subclassification as well as monitoring of disease activity. Indirect immunofluorescence (IIF) on HEp-2 (human epidermoid laryngeal carcinoma) cells has become the most established method for the screening of antibodies within the two-stage diagnostic strategy for SARD [4–6]. The unsurpassed high sensitivity of ANA assessment by IIF renders this method an ideal tool for the screening stage followed by confirmatory testing with different immunological assay technologies [4, 7, 8]. However, interpretation of IIF staining patterns is rather time consuming due to lacking automation and also highly subjective, making appropriate standardization difficult [4, 9]. Therefore, IIF has been increasingly replaced by novel techniques based on solid-phase immunoassays (e.g., ELISA, dot/line immunoassay, and addressable bead/microarray assays) [9–13]. These methods can be automated and are more cost efficient in particular in terms of the rising diagnostic demand due to the growing clinical impact of autoimmune diseases. However, high rates of false-negative findings have been reported for these techniques [10, 14]. Addressing this issue, the respective American College of Rheumatology (ACR) task force confirmed IIF as the gold standard for ANA testing [10].

Nevertheless, shortcomings of ANA assessment by IIF need to be overcome to employ this technique in a modern laboratory environment for SARD-associated antibody testing successfully. In the past decade, increasing standardization and automation efforts have been made to diminish the high intra- and interlaboratory variability and to render this method more accessible to high throughput screening [12, 15–18]. Apart from system solutions for automatic sample preparation, diagnostic companies have started to introduce new technologies for automated IIF pattern interpretation. These commercially available systems are generally based on digital acquisition and analysis of IIF images by pattern recognition algorithms. Some of these systems only distinguish between positive and negative screening results (Helios, Aesku.Diagnostics, Wendelsheim, Germany; Image Navigator, Immuno Concepts, Sacramento, USA; Cytospot, Autoimmun Diagnostika, Straßberg, Germany), whereas other systems are also able to classify basic staining patterns (AKLIDES, Medipan, Dahlewitz/Berlin, Germany; Nova View, Inova, San Diego, USA; Zenit G Sight, A. Menarini Diagnostics, Grassina-Firenze, Italy; Europattern, Euroimmun, Lübeck, Germany) [8, 19]. 

The fully automated interpretation system AKLIDES developed in the framework of the VideoScan technology is the first commercially available platform which has been evaluated in clinical studies [20, 21]. Based on fluorescence microscopy with different fluorochromes, the system is able to quantify fluorescence intensity and interpret basic staining patterns of HEp-2 cell IIF [22]. Recently, the application range of the AKLIDES platform has been expanded to ANCA and anti-double stranded DNA (dsDNA) AAb assessment employing fixed human neutrophils and *Crithidia luciliae *as substrates, respectively [23–25]. Furthermore, the AKLIDES system is now able to perform cell-based IIF assessment of *γ*H2AX foci used for individual biodosimetric evaluation of DNA double-strand breaks (DSBs) [26]. Remarkably, multiplex addressable microbead-based immunoassays (MIAs) have been developed for confirmatory testing of SARD-associated antibodies on the AKLIDES platform thereby creating the first combined diagnostic solution for IIF screening and confirmatory testing in autoimmune serology. 

 The present paper provides an overview of recently published studies comparing the AKLIDES system with methods used in routine diagnostics referring to standardization, automation, and reliability of this new technology. 

## 2. AKLIDES Platform

### 2.1. Technical System and Composition

The AKLIDES system is based on a novel composition of different hardware modules combined with innovative mathematical pattern recognition software algorithms, enabling fully automated image acquisition, analysis, and evaluation of immunofluorescence tests. 

**Figure 1:**
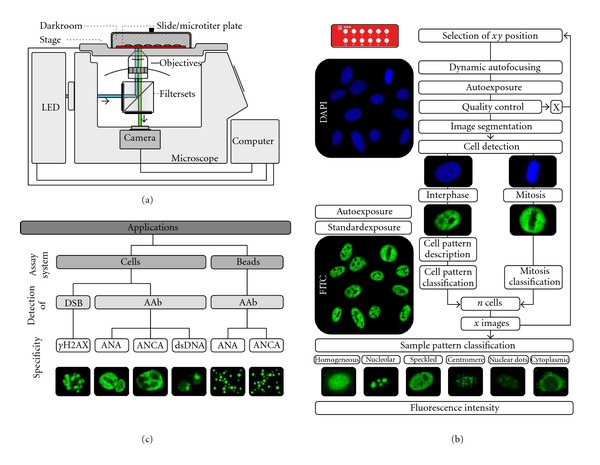
AKLIDES platform for automated evaluation of cell-based IIF testing and multiplex addressable microbead-based immunoassays analysis. (a) Schematic drawing of the main hardware components of the AKLIDES system based on a fluorescence microscope combined with different filtersets and objectives, a LED unit, a gray level camera, a movable scanning stage, and a controlling computer. (b) Flowchart of measurement and evaluation of automated HEp-2 IIF assay interpretation by the AKLIDES system including a sequential, multistage process of image acquisition, quality control, object segmentation, object description, and object classification [27]. (c) Application range of the AKLIDES system divided into evaluation of cell-based IIF testing for DSB and AAb detection as well as AAb analysis using bead-based multiplex assays.

The backbone of the AKLIDES system is formed by a motorized inverse fluorescence microscope (Olympus IX81, Olympus Corp., Tokyo, Japan) including different objectives and dualband filtersets, which can be switched automatically. A movable scanning stage (IM120, Märzhäuser, Wetzlar, Germany) with exchangeable inlays allows precise selection of the requested xy position and measurement of slides as well as microtiter plates. Fluorescence excitation is achieved through light emitting diodes (LEDs) (precisExcite, CoolLED, Hampshire, UK). A gray level camera (PS4, Kappa opto-electronics, Göttingen, Germany) is used for image acquisition. All devices are connected to a computer to run necessary software applications and to provide sufficient data storage (Figure 1(a)). The software contains modules to control hardware equipment, the software autofocus, image analyzing algorithms, and data analysis. All algorithms were implemented using programming language C++ (Visual Studio 2008; Microsoft, Redmond, USA) and OpenMP for parallelization of tasks. For automatic image acquisition a novel software-based autofocus was developed based on Haralick's characterization of image texture by analyzing occurrences of gray scale transition. Additional nuclear staining with 4′,6-diamidino-2-phenylindole (DAPI) within cell based assays was used for autofocusing, quality evaluation, and object recognition [22, 27].

### 2.2. Software Concept

Automatic IIF evaluation by AKLIDES comprises a sequential, multistage process including image acquisition, quality control, object segmentation, object description, and object classification (Figure 1(b)). After selecting the requested xy position on the slide, dynamic autofocusing is performed in DAPI channel, starting with coarse focusing to find the approximate position of the substrate, subsequently followed by fine focusing with narrow z steps to determine the exact focal plane [28]. To exclude images that are not suitable for further evaluation due to over- or underexposure, artifacts, air entrapments, or inhomogeneities in fluorescence staining, a qualitative image analysis was implemented. Therefore, every image is divided into tiles of equal size. Comparison of subsequently calculated tile sharpness, and homogeneity was used for quality evaluation [22]. In order to select circular-shaped elements in the images (cell nuclei, beads), object segmentation was accomplished by using a histogram-based mixture model threshold algorithm to model the background intensity, followed by watershed transformation. Segmented objects were characterized by different boundary, regional, topological, and texture/surface descriptors. For pattern recognition, 200 attributes were implemented, leading to a variety of approximately 1,400 object describing criteria after appropriate combination [22].

DAPI staining was used to detect cell location and to identify mitotic cells, whereas pattern recognition was performed in FITC channel mode. Object classification of immunofluorescence patterns was accomplished by combining structural and textural features following a sequence of three hierarchical decision steps: (i) positive/negative discrimination, (ii) location assessment of nucleus, cytoplasm, and chromatin of mitotic cells, and (iii) fluorescence pattern recognition [27]. With the current approach six basic HEp-2 cell staining patterns can be distinguished: cytoplasmic, homogeneous, speckled, nucleolar, centromere, and multiple nuclear dots [29]. Automatic pattern recognition can be achieved by two different strategies: self-learning algorithms and those using static classificators. AKLIDES is based on static classificators in order to standardize interpretation of HEp-2 assays, whereas self-learning methods would increase interlaboratory variance [29].

For image intensity evaluation, the AKLIDES software calculates a reactivity index (RI), combining several parameters including image intensity, image contrast, and number of gray level occurring in the entire image. The RI is also influenced by the exposure time. To ensure an ideal exposure, an automatic computing method was implemented to correct the exposure time of the scene depending on the highest gray level value in the image (single artifacts excluded). This even allows the detection of patterns with weak absolute image intensity (e.g., centriole, multiple nuclear dots). On the basis of the RI value of 200 blood donor samples cut-offs for borderline and positive reactivities were determined [27, 29].

Evaluation of multiplex addressable MIA is comparable to already described cell assay except for DAPI counter staining and detection of subcellular locations. Essential autofocusing and quality control are directly performed with polymerized microbeads, and substructuring of microbead surface is not required.

### 2.3. Applications

To overcome the drawbacks of IIF and to address the increasing demand for SARD-associated antibody detection, different technical approaches for automated IIF testing have been developed [8, 15, 16, 18, 21, 29].

Based on fluorescence intensity quantification and pattern recognition of IIF images, commercially available IIF assays for ANA and ACyA have been introduced on the fully automated IIF interpretation system AKLIDES [20, 23, 25, 30]. Antibody detection and fluorescence staining are performed using fluorescein-isothiocyanate-coupled sheep anti-human IgG conjugate and DAPI-containing mounting medium. After adaptation of pattern recognition algorithms, the application spectrum of the AKLIDES system was expanded to ANCA detection on formalin and ethanol fixed human neutrophil granulocytes as well as detection of AAb against dsDNA using *Crithidia luciliae* immunofluorescence tests (CLIFTs). 

By incorporating addressable MIA for multiplexing, the application range of the AKLIDES platform presents a unique system solution for SARD serology and can be divided into two major groups, respectively, (i) screening of antibodies by cell-based IIF assays and (ii) analyzing of multiplexed microbead-based immunoassays as confirmatory testing for AAb detection. A further novel application of the AKLIDES system is the measurement of dsDNA DSBs by evaluating *γ*H2AX foci (Figure 1(c)).

## 3. Screening of AAb by Cell-Based Assays

### 3.1. ANA Detection on HEp-2 Cells

In 2010, Egerer et al. reported the first clinical evaluation of an automated IIF interpretation system by using AKLIDES technology for ANA assessment in the routine laboratory environment of both a university and a private referral laboratory [20]. Comparing positive and negative findings of 1,222 sera obtained from patients with suspected SARD, an agreement of 93.0% (859/924) and of 90.6% (270/298) between AKLIDES interpretation and visual reading of ANA in the university and the private laboratory was achieved, respectively. Further pattern evaluation comparing visually and automatically defined fluorescence patterns of AKLIDES positive samples revealed an agreement of 90.1% and 92.7% for the university and private laboratory, respectively. Discrepancies in image recognition were mainly seen for sera demonstrating mixed patterns, AAb against nuclear membrane or cytoplasmic staining [20].

Recent studies by Kivity et al. and Melegari et al. showed similar results [30, 31]. In the report by Kivity et al., ANA and ACyA assessment by AKLIDES was compared to manual screening on HEp-2 cells and to an ANA immunodot assay. A total of 397 sera samples were investigated, including 137 apparently healthy donors, 34 patients with SLE, 111 patients with DM or PM, 74 patients with SSc, and in addition 41 samples with rare AAb pattern (e.g., nuclear dots, Golgi apparatus, lysosomal like). There was 100% agreement among the 34 SLE samples, which were all tested positive in the three methods applied. The AKLIDES system detected more positive results in DM/PM (95%) compared to manual HEp-2 cell interpretation (74%) and immunodot assay (64%). Out of 74 sera from patients with SSc 97% were tested positive by AKLIDES, whereas manual reading of HEp-2 cells and immunodot analysis only showed positive findings in 92% and 86% of these cases, respectively. According to ROC curve analysis for the interpretation of DM/PM and SSc sera using the AKLIDES system, sensitivities between 97-98% and specificities between 91-92% could be calculated. Regarding the analysis of SSc sera, pattern recognition was found to be correct in 82% of anti-CENP-B-positive samples and in 72% of anti-Scl-70 positives. Analysis of 41 sera with rare AAb by the AKLIDES system demonstrated positive findings in 95% of the cases investigated and showed a good correlation between manual and automated pattern recognition in terms of distinguishing between nuclear and cytoplasmic staining. A further study reported by Melegari et al. also confirmed the excellent agreement of positive and negative findings by visual and AKLIDES reading of IIF on HEp-2 cells [30]. Analyzing 66 routine samples and 116 selected samples with known AAb levels, only two discordant findings were obtained with the AKLIDES system, resulting in a remarkable total agreement for ANA screening of 98.9%. 

### 3.2. ANCA Detection on Human Neutrophil Granulocytes

Besides ANA testing also ANCA assessment by the AKLIDES system was evaluated by Melegari et al. [30]. In this study, 46 samples were analyzed by IIF on the AKLIDES system, by two experts on a fluorescence microscope as well as by anti-MPO and anti-PR3 ELISA. The agreement between manual and automated IIF reading was reported to be 89.1% (41/46).

In a different study by Knuetter et al., ANCA testing was performed in 293 sera from patients with AASV and other SARDs on ethanol- and formalin-fixed neutrophils [23]. Comparison of positive and negative findings between manual and automatic reading revealed a very good agreement for ethanol- (*κ* = 0.871) and formalin-fixed neutrophils, (**κ** = 0.866). Furthermore, differentiation of cANCA and pANCA pattern by the AKLIDES system showed a good agreement for ethanol- (*κ* = 0.739) and formalin-fixed neutrophils (**κ** = 0.742) [23].

In a recent report by Damoiseaux et al. comparing visual and automated ANCA evaluation of ethanol- and formalin-fixed neutrophils, sera from patients with AASV tested positive for MPO- (*n* = 40) or PR3-ANCA (*n* = 39), and different groups of control sera were analyzed [24]. Visual IIF testing of PR3-ANCA-positive patients showed a cANCA pattern in 92% of the cases on ethanol- and in 97% on formalin-fixed slides, whereas AKLIDES reported positive cANCA findings in 74% and 95% of the samples, respectively. Concerning ethanol-fixed neutrophils, 90% of sera from MPO-ANCA-positive patients revealed a pANCA pattern using routine microscopy, whereas AKLIDES detected pANCA staining in 80% of the samples [24].

### 3.3. Anti-dsDNA AAb Detection on *Crithidia luciliae*

Anti-dsDNA antibodies can be readily detected by IIF employing CLIFT [32, 33]. Automated interpretation of CLIFT by AKLIDES provides the basis for the standardized detection of highly disease-specific anti-dsDNA antibodies. In a recent study including 44 serum samples of SLE patients, Melegari et al. compared automated and visual analysis of IIF approaches for anti-dsDNA antibody detection on *Crithidia luciliae* as well as corresponding ELISA data. The agreement between the results obtained by expert reading, and the AKLIDES system was 91.0% [30].

## 4. Confirmatory Testing of AAb by Multiplexed Addressable Microbead-Based Assays

Positive antibody findings in the screening for SARD serology are recommended to be confirmed by molecular immunoassays in a second diagnostic stage. In order to create a combined platform allowing both high-sensitive antibody screening by IIF and analysis of specific AAb, MIAs were developed for the AKLIDES system. These addressable MIAs utilize multiple carboxylated polymethylmethacrylate bead populations differing in size and/or concentrations of fluorescent dye for multiplexing. Each population was covalently labeled with a specific antigen, and beads were immobilized onto 96-well microtiter plates. By suspending different bead populations within one well, a multiplex assay can be performed. Bound AAb were detected with secondary fluorophore-coupled anti-human-IgG antibodies. Classification of bead populations and measurement of corresponding ligand fluorescence intensity can be readily performed by AKLIDES [34].

### 4.1. ANA Multiplex Assay

A multiplex MIA for detection of six different antinuclear AAb on the AKLIDES system was developed and evaluated by Grossmann et al. [34]. In total, seven microbead populations differing in size and/or ratio of fluorescent dyes were covalently labeled with either Scl-70, Sm, SS-A (Ro60), SS-B (La), CENP-B, dsDNA or human IgG. Bead classification and measurement of mean fluorescence intensity of 72 sera from patients with autoimmune diseases were performed by the AKLIDES systems. For comparison of results obtained by MIA and ELISA, Cohen's kappa was determined, showing perfect agreement concerning Scl-70, Sm, CENP-B, and SS-B AAb (*κ* = 1.000). A very good agreement was found for dsDNA (*κ* = 0.961) and good agreement for SS-A (*κ* = 0.783) AAb, respectively [34].

### 4.2. ANCA Multiplex Assay

In order to accomplish specific antibody assessment for the serological diagnosis of AASV, a multiplex addressable MIA was developed, enabling detection of AAb against MPO, PR3, and the noncollagen region of the alpha-3 subunit of collagen IV (GBM). 

Addressable MIA was performed of 265 sera, including 51 patients with active granulomatosis with polyangiitis (Wegener's), 41 patients with microscopic polyangiitis, and 10 patients with Goodpasture syndrome (GPS) or anti-GBM nephritis as well as 108 control sera from donors with ANA-associated systemic rheumatic disease and 55 donors with rheumatoid arthritis. Results of MIA were compared with results achieved by IIF and ELISA. Comparison of MIA findings by AKLIDES with results of conventional assays showed a very good interrater agreement based on Cohen's kappa calculation for anti-PR3 (*κ* = 0.927), anti-MPO (*κ* = 0.868), and anti-GBM testing (*κ* = 0.938). Discrepancies detected between the different methods were mainly found in sera with low reactivities [34].

## 5. Detection of Double-Strand Breaks by Automated Indirect Immunofluorescence Testing

The image processing capabilities and fluorescence pattern recognition algorithms of the AKLIDES system represent a platform beyond antibody detection by IIF. One further application is the quantification of dsDNA DSB. After DSB formation, large numbers of histone H2AX molecules in the vicinity of the DSB become phosphorylated at serin139 (*γ*H2AX), leading to complex formation including different molecules responsible for DNA repair and chromatin remodeling. The number of fluorescent foci revealed by specific anti-*γ*H2AX staining of these particular complexes has been found to correlate with the number of DSB [35, 36]. Quantification can be performed by fluorescence microscopy, determining the average number of fluorescent *γ*H2AX found in the nuclei of 100 cells [26]. Since visual interpretation is time consuming and heavily influenced by subjective reading, the AKLIDES system was adapted for fully automated assessment of *γ*H2AX foci.

Results of automated interpretation of radiation induced *γ*H2AX foci were reported by Runge et al. in 2012 [26]. In this study, *γ*H2AX analysis was performed by manual reading of three laboratories and by fully automated AKLIDES measurement investigating PCCl3 cells (thyroid rat cell line) after exposure to different doses (0–5 Gy) of ^188^Re. Data confirmed a high interlaboratory variability of 38.4% for manual reading of *γ*H2AX foci assessment in different laboratories. In contrast, a good agreement of automated and manual *γ*H2AX foci analysis was revealed between AKLIDES and the laboratory which collaborated in the adaption of the pattern recognition algorithms for AKLIDES (*R* ^2^ = 0.931) [26].

## 6. Conclusion

Testing for AAb is an essential step in the serological diagnosis of autoimmune diseases in particular SARD [3, 4, 37]. As a matter of fact, one of the first techniques available in routine laboratories and still the recommended method for ANA screening is IIF testing currently preferably on HEp-2 cells [38]. However, the IIF technique is characterized by time-consuming and subjective evaluation, insufficient automation as well as poor standardization [11, 39]. Furthermore, inconsistencies in description and classification of staining patterns have been reported to hamper standardization efforts [9, 20, 31, 40]. To respond to the growing number of particularly ANA tests for SARD serology, new methods based on solid phase immunoassays, like ELISA or multiplexing technologies have been developed [13, 41–46]. However, ANA testing by IIF is still recommended to be used as the gold standard method due to its high sensitivity and may outperform microbead technology under optimal conditions [10, 47]. Employing a limited number of autoantigens, ANA ELISA or ANA multiplex assays revealed up to 35% false-negative results compared with ANA IIF testing on HEp-2 cells [10]. In order to automate and standardize ANA detection on HEp-2 cells, different commercially available platforms were developed, and preliminary data show high agreement between visual and automated ANA interpretation [8, 17, 19, 27, 48, 49]. In particular, progress in automated interpretation of subcellular patterns and location determination as well as novel pattern recognition algorithms for IIF has pioneered the commercialization of this new technology [50–52].

Recently, several studies have been published about the performances of the first fully automated interpretation system AKLIDES, combining automatic image acquisition and pattern recognition. The AKLIDES platform has been developed to become a multipurpose bioanalytical tool, which is able to analyze different kinds of cell- and bead-based fluorescence assays [22, 27, 28]. Reported data showed a high agreement between manual and automated interpretation of cell-based IIF testing for ANA, ANCA, and anti-dsDNA assessment. Not only automated discrimination between positive and negative results but also pattern recognition of fluorescence images showed a good correlation with visual IIF reading. Although the number of patterns recognized and pattern recognition accuracy needs to be further improved, automatic interpretation of cell-based IIF assays by AKLIDES may be used in autoimmune laboratories and can be a helpful screening tool in routine diagnostics especially for exclusion of negative samples [31]. Nevertheless, positive findings provided by the system should always be confirmed by an expert [30].

Most clinical immunology laboratories apply a two-stage approach for ANA testing, starting with an initial screening on HEp-2 cells, which is followed by confirmatory testing [3, 4, 11]. Expanding the AKLIDES systems to assess addressable MIA created a unique platform, which for the first time allows fully automated evaluation of cell-based screening tests and antigen-specific multiplex assays on one system. Detecting multiple SARD-associated AAb simultaneously in one run, multiplex ANA testing is more efficient compared to the conventional ELISA technique. With regard to most of the investigated autoantigens, studies demonstrated very good agreements of results achieved with either ANA or ANCA multiplex assay and corresponding single ELISAs. Thus, multiplexing provides the basis for antibody profiling as an efficient and promising approach in the serology of autoimmune disorders [53–57].

The technology of digital fluorescence image acquisition and automatic pattern recognition can be extended to other currently established cell-based IIF assays. Such a different cell-based IIF technique is the quantification of *γ*H2AX foci, which is very time consuming, subjective, and not suitable for high-throughput screening in its present version [26]. Besides satisfying performances regarding SARD-associated antibody assessment, the AKLIDES system demonstrated adequate results for the automated assessment of *γ*H2AX foci in irradiated cells. 

The need for both standardized SARD-associated antibody detection as well as evaluation of *γ*H2AX foci is growing [26, 40, 58]. The AKLIDES technology can help to improve inter- and intralaboratory variations and may enable high-throughput diagnostics. Furthermore, cost-efficient analysis of large volumes of samples in routine laboratories may support the finding of new relevant antibodies for improved antibody profiling in SARD serology [3].

## Abbreviations

AAb: Autoantibody
ANA: Antinuclear antibody
ACyA: Anticytoplasmic antibody
ANCA: Antineutrophil cytoplasmic antibody
AASV: ANCA-associated systemic vasculitis
cANCA: Cytoplasmic ANCA
CLIFT: *Crithidia luciliae* immunofluorescence test
DSB: Double-strand break
dsDNA: Double-stranded DNA
ELISA: Enzyme-linked immunosorbent assays
GBM: Antiglomerular basement membrane
GPS: Goodpasture syndrome
IIF: Indirect immunofluorescence
IIM: Idiopathic inflammatory myopathy
LED: Light-emitting diode
MIA: Microbead-based immunoassay
MPO: Myeloperoxidase;
pANCA: Perinuclear ANCA
PR3: Proteinase 3
RA: Rheumatoid arthritis
SARD: Systemic autoimmune rheumatic disease
SLE: Systemic lupus erythematosus
SjS: Sjögren's syndrome
SSc: Systemic sclerosis.
